# Beyond PARP1: The Potential of Other Members of the Poly (ADP-Ribose) Polymerase Family in DNA Repair and Cancer Therapeutics

**DOI:** 10.3389/fcell.2021.801200

**Published:** 2022-01-14

**Authors:** Iain A. Richard, Joshua T. Burgess, Kenneth J. O’Byrne, Emma Bolderson

**Affiliations:** ^1^ Cancer and Ageing Research Program (CARP), Centre for Genomics and Personalised Health (CGPH), Queensland University of Technology (QUT), Brisbane, QLD, Australia; ^2^ Princess Alexandra Hospital, Brisbane, QLD, Australia

**Keywords:** PARP, cancer, DNA damage, DNA repair, genomic stability, tumourigenesis

## Abstract

The proteins within the Poly-ADP Ribose Polymerase (PARP) family encompass a diverse and integral set of cellular functions. PARP1 and PARP2 have been extensively studied for their roles in DNA repair and as targets for cancer therapeutics. Several PARP inhibitors (PARPi) have been approved for clinical use, however, while their efficacy is promising, tumours readily develop PARPi resistance. Many other members of the PARP protein family share catalytic domain homology with PARP1/2, however, these proteins are comparatively understudied, particularly in the context of DNA damage repair and tumourigenesis. This review explores the functions of PARP4,6-16 and discusses the current knowledge of the potential roles these proteins may play in DNA damage repair and as targets for cancer therapeutics.

## Introduction

As global populations age, cancer has emerged as the most prominent cause of death worldwide (Lin et al., 2019; Aburto et al., 2020; Sung et al., 2021). Therefore, identifying new therapeutic targets and designing non-invasive molecular mechanisms to inhibit and eliminate cancer growth is a major objective of academic and pharmaceutical teams worldwide. One such protein that has become a new therapeutic cancer target in recent years is Poly (ADP-ribose) Polymerase 1 (PARP1), which belongs to the PARP protein family. The members of this protein family have been associated with DNA repair, genomic instability and as targets for cancer therapy (D’Amours et al., 1999; Amé et al., 2004; Berti et al., 2013; Morales et al., 2014; Schlacher, 2017). Supporting this, PARP1 has emerged as a potent cancer target in ovarian and breast cancers. Since much is known about PARP1-3 (Bryant et al., 2005; Farmer et al., 2005; Murai et al., 2012; Ray Chaudhuri and Nussenzweig, 2017; Alemasova and Lavrik, 2019; Rodriguez-Vargas et al., 2019; Bilokapic et al., 2020) and the Tankyrases (PARP5a/5b) (Lakshmi et al., 2017; Li et al., 2019), this review will focus on the lesser-studied PARP family members, their roles in maintenance of genomic stability and cellular homeostasis, and their potential as cancer targets (Hottiger et al., 2010; Morales et al., 2014; Xu et al., 2020; Challa et al., 2021).

## The PARP Family

The PARP protein family was initially described in 1963 (Chambon et al., 1963) and the crystal structure of the PARP1 catalytic domain was later elucidated in 1996 (Ruf et al., 1996). This unique family consists of 17 proteins to date (Amé et al., 2004; Hottiger et al., 2010; Morales et al., 2014; Challa et al., 2021), excluding the highly diverged PARP homologue tRNA 2′-phosphotransferase 1 (TRPT1) (Hottiger et al., 2010). The full range of functionality of this protein family has not been fully elucidated, however, they have all been shown (with the exception of PARP13) to catalyse the transfer of ADP-ribose (Morales et al., 2014) to substrates, via the use of nicotinamide adenine dinucleotide (NAD+) as a metabolic substrate (Hottiger et al., 2010; Morales et al., 2014; Gupte et al., 2017; Cohen, 2020). This modification is referred to as ADP ribosylation (ADPr). ADPr has so far been identified as important in many cellular processes, including transcription, chromatic structure modulation, replication, recombination, and DNA damage repair (D’Amours et al., 1999; Morales et al., 2014).

PARPs fall into two main categories depending on the (ADPr) modification they produce. These categories are mono-[ADPr] (MAR), and Poly-[ADPr] (PAR) (Figure 2). This difference is mechanistically important in biological processes. PAR ribosylation modifications create branched elongated chains that commonly act as signaling molecules (Ruf et al., 1996; Amé et al., 2004; Hottiger et al., 2010; Vyas et al., 2013). Current studies indicate that only PARPs with a H-Y-E amino acid triad domain can produce PAR modifications, due to the glutamic acid residue (E) facilitating the process of producing these elongated ribosylation chains (Hottiger et al., 2010; Challa et al., 2021). However, it is important to note that PARP3 contains a H-Y-E domain motif, but does not produce PAR chains, suggesting that the motif is not the only structural driving factor of PARylation (Hottiger et al., 2010; Vyas et al., 2014; Challa et al., 2021). There is less data surrounding the functional importance of MAR modifications. However, MAR modifications typically inhibit target protein function, which suggests a direct regulatory role (refer to Table 1 for PARP specific catalytic activity). Despite this fundamental difference, both PAR and MAR modifications utilise NAD+ as a substrate (Corda and Di Girolamo, 2003; Hottiger et al., 2010; Cohen, 2020). The PARP protein family is also involved in the formation of non-membranous structures (Amé et al., 2004; Vyas et al., 2013; Catara et al., 2017; Challa et al., 2021). These structures include: spindle poles, RNA granules, and DNA repair foci (Catara et al., 2017).

**TABLE 1:** Overview of PARP family structure and basic function.

Name	Other names	Molecular weight (Da)	Amino acid length	Catalytic triad sequence	Type of ribosylation activity (PAR or MAR)	DNA dependent activation	Inhibitors available—FDA approval status
PARP1	PARP, ARTD1	113,084	1,014	H-Y-E Hottiger et al. (2010), Challa et al. (2021)	PAR Ko and Ren (2012)	Yes De Vos et al. (2012), Vyas et al. (2013)	Yes—Approved for prostate cancer, breast cancer, ovarian cancer and gynecologic cancer. Sisay and Edessa (2017), Dal Molin et al. (2018), Cortesi et al. (2021)
PARP2	ARTD2	66,206	583	H-Y-E Hottiger et al. (2010), Challa et al. (2021)	PAR Ali et al. (2016)	Yes De Vos et al. (2012), Ali et al. (2016)	Yes - Approved for prostate cancer, breast cancer, ovarian cancer and gynecologic cancer. Sisay and Edessa (2017), Dal Molin et al. (2018), Cortesi et al. (2021)
PARP3	ARTD3	60,089	533	H-Y-E Hottiger et al. (2010), Challa et al. (2021)	MAR Rodriguez-Vargas et al. (2019), Challa et al., 2021)	Yes De Vos et al. (2012)	Yes—Approved for ovarian cancer Sisay and Edessa (2017), Dal Molin et al. (2018)
PARP4	vPARP, ARTD4	37,288	327	H-Y-E Hottiger et al. (2010), Challa et al. (2021)	MAR (PAR when localised to vault particles) Kickhoefer et al. (1999); Challa et al., 2021)	No	Yes—Not FDA approved Dal Molin et al. (2018), Kirby et al. (2021)
PARP5a	TNKS1, ARTD5	142,039	1,327	H-Y-E Hottiger et al. (2010), Haikarainen et al. (2014)	PAR Haikarainen et al. (2014)	Postulated (De Vos et al. (2012), Haikarainen et al. (2014)	Yes—Not FDA approved. Sisay and Edessa (2017), Dal Molin et al. (2018), Cortesi et al. (2021)
PARP5b	TNKS2, ARTD6	126,918	1,166	H-Y-E Hottiger et al. (2010), Haikarainen et al. (2014)	PAR Haikarainen et al. (2014)	Postulated De Vos et al. (2012), Haikarainen et al. (2014)	Yes—Not FDA approved. Sisay and Edessa (2017), Dal Molin et al. (2018), Cortesi et al. (2021)
PARP6	ARTD17	71,115	630	H-Y-I Hottiger et al. (2010), Challa et al. (2021)	MAR Challa et al. (2021)	Undetermined	Yes—Not FDA approved. Wang et al. (2018)
PARP7	tiPARP, ARTD14	76,227	657	H-Y-I Hottiger et al. (2010), Challa et al. (2021)	MAR Challa et al. (2021)	Undetermined	Yes—Not FDA approved. Gozgit et al. (2021)
PARP8	ARTD16	95,871	854	H-Y-I Hottiger et al. (2010), Challa et al. (2021)	MAR Challa et al. (2021)	Undetermined	No
PARP9	BAL1, ARTD9	96,343	854	Q-Y-T (Hottiger et al., 2010; Xu et al., 2020; Xing et al., 2021)	MAR Yang et al. (2017)	Undetermined	No
PARP10	ARTD10	109,998	1,025	H-Y-I Hottiger et al. (2010), Challa et al. (2021)	MAR Challa et al. (2021)	No Vyas et al. (2013)	Yes—Not FDA approved.Lemke et al. (2020)
PARP11	ARTD11	39,597	338	H-Y-I Hottiger et al. (2010), Challa et al. (2021)	MAR Challa et al. (2021)	Undetermined	Yes—Not FDA approved. Kirby et al. (2018)
PARP12	ARTD12	79,064	701	H-Y-I Hottiger et al. (2010), Challa et al. (2021)	MAR Challa et al. (2021)	Undetermined	Yes—(Nonselective)—Not approved for PARP12. Dal Molin et al. (2018)
PARP13	ZAP, ARTD13	101,431	902	Y-Y-V Hottiger et al. (2010), Morales et al. (2014), Challa et al. (2021)	Catalytically Inactive—MAR Postulated Hottiger et al. (2010), Morales et al. (2014), Challa et al. (2021)	Undetermined	No
PARP14	BAL2, ARTD8	202,800	1,801	H-Y-L Hottiger et al. (2010), Challa et al. (2021)	MAR Challa et al. (2021)	Undetermined	Yes—Not FDA approved. Schenkel et al. (2021)
PARP15	BAL3, ARTD4	74,576	678	H-Y-L Hottiger et al. (2010), Challa et al. (2021)	MAR Challa et al. (2021)	Undetermined	Yes (Nonselective) - Not FDA approved for PARP15. Dal Molin et al. (2018)
PARP16	ARTD15	36,383	332	H-Y-Y Hottiger et al. (2010), Challa et al. (2021)	MAR Challa et al. (2021)	Undetermined	Yes (Nonselective)—Not FDA approved for PARP16. Sisay and Edessa (2017), Dal Molin et al. (2018), Cortesi et al. (2021), Palve et al. (2021)

Molecular Weight and Amino Acid Length were derived from UniProt database

PARP1-3 have been identified as regulatory proteins in single-strand break repair pathways (Fisher et al., 2007; Hanzlikova et al., 2018; Rose et al., 2020). In recent years PARP inhibitors (PARPi) have been developed as a novel targeted cancer therapeutic (Dziadkowiec et al., 2016; Rose et al., 2020). These inhibitors work on tumours that are deficient in the double-strand break repair pathway of homologous recombination, caused by the dysfunction of proteins such as *BRCA1/2*, via promotor methylation or gene mutation (Dziadkowiec et al., 2016; Rose et al., 2020). These defects can be used to target tumours using PARPi, that bind to the NAD+ binding domain of several PARPs, predominantly PARP1/2, inhibiting their catalytic activity and trapping them on DNA (Dziadkowiec et al., 2016; Ronson et al., 2018; Rose et al., 2020). This inhibition can be used as a selective target in *BRAC1/2* deficient cancer cells leading to a buildup of highly cytotoxic unrepairable double-strand breaks, resulting in cell death. This review will examine the potential of other members of the PARP family as targets for cancer therapy.

## The Roles of the PARP Family in Cellular Homeostasis: Implications for Tumourigenesis and Cancer Therapy

**FIGURE 1:**
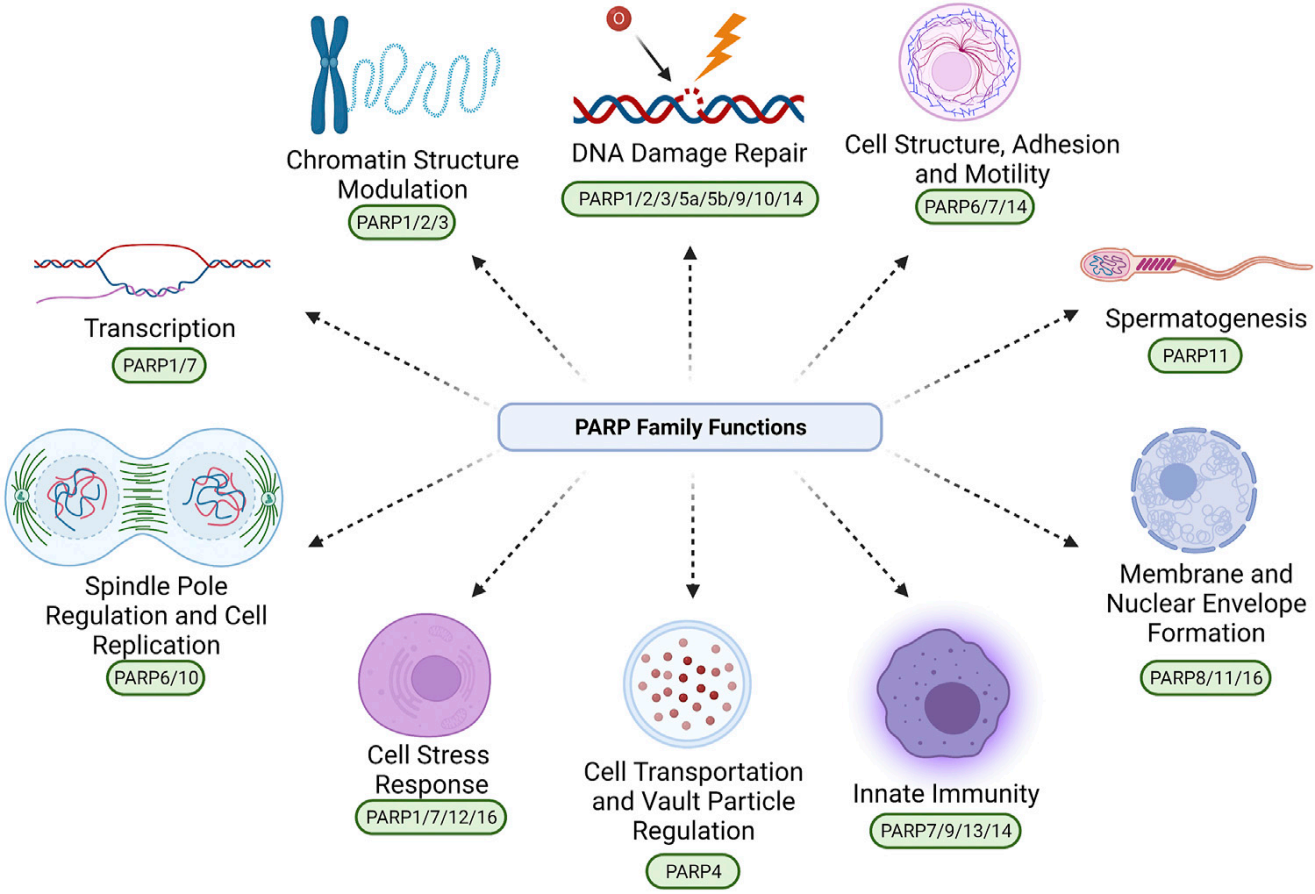
The confirmed and proposed diverse roles of the PARP protein family. These roles include: DNA Damage Repair (PARP1/2/3/5a/5b/9/10/14), Cell Structure, Adhesion and Motility (PARP6/7/14), Spermatogenesis (PARP11), Membrane and Nuclear Envelope Formation (PARP8/11/16), Innate Immunity (PARP7/9/13/14), Cell Transportation and Vault Particle Regulation (PARP4), Cell Stress Response (PARP1/7/12/16), Spindle Pole Regulation and Cell Replication (PARP6/10), Transcription (PARP1/7), and Chromatin Structure Modulation (PARP1/2/3). Due to the large diversity of PARP activity, it is likely that PARPs are also involved in biological processes beyond those exemplified in this figure, that are yet to be fully elucidated. Created with BioRender.com.

**FIGURE 2:**
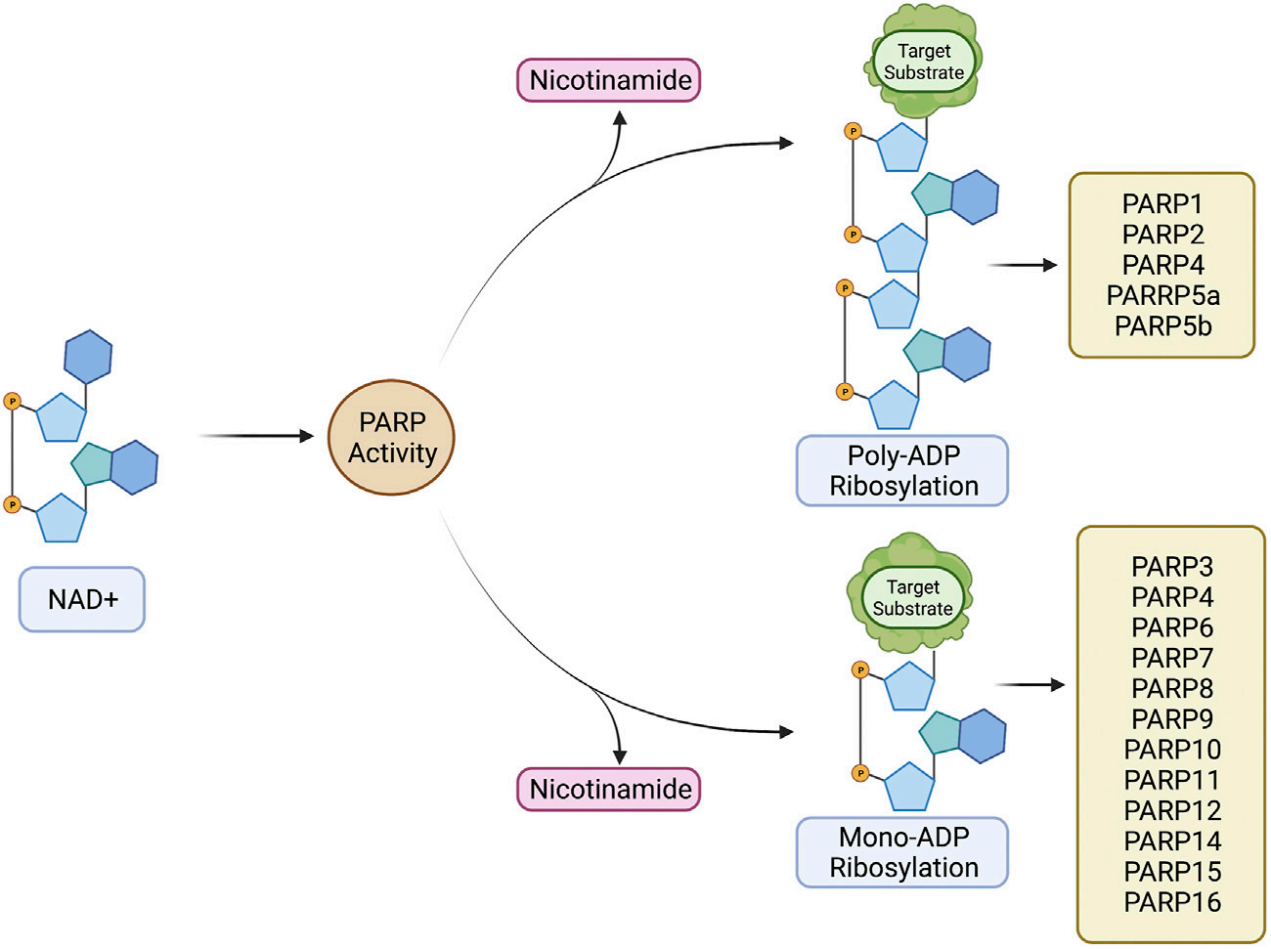
PARP family-dependent poly and mono ADP ribosylation. These processes comprise conversion of NAD+ to a ribosylation modification via PARP catalytic activity, producing nicotinamide as a biproduct. PARP1/2/4/5a/5b have been experimentally shown to produce poly-ADP ribosylation modifications. PARP3/4/6/7/8/9/10/11/12/14/15/16 have been experimentally shown to produce mono-ADP ribosylation modifications. Created with BioRender.com.

The proteins within the PARP family function to maintain cellular homeostasis through their involvement in a diverse array of biological pathways, beyond DNA damage repair (Figure 1). Through these diverse pathways the upregulation, depletion or mutation of these unique proteins can promote tumourigenesis. Although their catalytic domains share homology, PARP proteins vary widely in size and structure promoting a rich diversity of functions. PARPs range from 36.38 kDa (PARP16) to 202.8 kDa (PARP14) in size (Table 1). Their catalytic activity also varies, PARP1-2/4/5a/5b produce PAR modifications, whereas PARP3/4/6-12/14-16 produce MAR modifications (Figure 2; Table 1).

**FIGURE 3:**
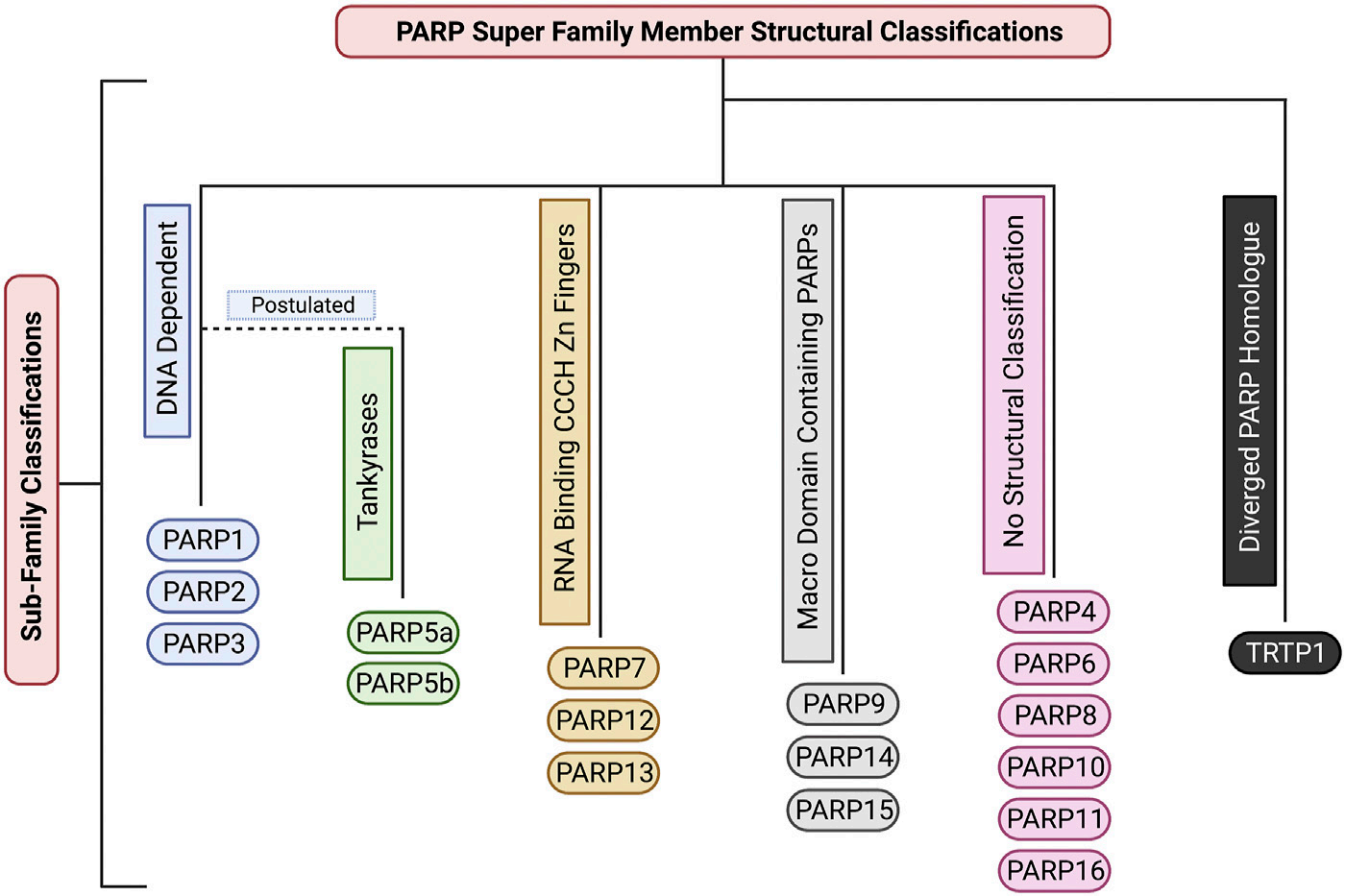
The structural sub-classifications of the PARP family. These classifications include: DNA Dependent PARPs (PARP1/2/3), Tankyrases (PARP5a/5b), RNA Binding CCCH Zn Finger PARPs (PARP7/12/13), Macro Domain Containing PARPs (PARP9/14/15), PARPs with no sub-classification (PARP4/6/8/10/11/16), and Diverged PARP Homologues (TRTP1). Created with BioRender.com.

Due to its MARylation activity (Figure 2; Table 1), PARP4 has been categorized as a mono-[ADP-ribosyl] transferase (MART), however, PARP4 is not currently included in any PARP sub-family classification (Figure 3). Notably, this MART classification is true despite PARP4 having a H-Y-E catalytic triad domain which is commonly associated with PARylation (Vyas et al., 2013; Challa et al., 2021). Being a MART, it is likely that PARP4 is involved in protein regulation and transport (Challa et al., 2021). Supporting this, a study has implicated PARP4 in the regulation of vault ribonucleoprotein particle function (Kickhoefer et al., 1999). Interestingly, PARP4 begins producing PAR chains after re-localising to vault particles (Kickhoefer et al., 1999; Vyas et al., 2014). Vault particles are comparatively large highly conserved biological structures, comprising of hollow barrel structures, around 13 MDa in size, that are believed to be involved in intracellular transport of materials (Kickhoefer et al., 1999; Mossink et al., 2003; Woodward et al., 2015). In relation to cancer, a study found that two PARP4 mutations were found in 43% of their cohort diagnosed with breast and thyroid cancer (Ikeda et al., 2016). Conversely, these mutations were only present in 0.5% of the control cohort. Low PARP4 levels were also associated with poorer prognosis (Ikeda et al., 2016). This suggests that PARP4 may have a role in suppressing tumourigenesis.

Although several of the clinically approved PARP inhibitors also target PARP4, in addition to PARP1-3, it is unclear what the effect of inhibiting PARP4 would have on tumour cells. However, a recent study describes the rational design of a new inhibitor to target PARP4 via its unique threonine residue in the nicotinamide sub-pocket (Kirby et al., 2021). The study found that the AEP07 compound had a 12-fold selectivity for PARP4 over other PARP family members and may form the basis for the further investigation of the activity and development of specific PARP4 inhibitors for therapeutic applications (Kirby et al., 2021).

Like PARP4, PARP6 produces MAR modifications (Figure 2; Table 1). Due to its unique structure it currently does not belong to any sub-family classification (Figure 3). However, recent studies have elucidated that PARP6 is involved in some key cellular functions. One study showed that PARP6 enzyme inhibition induces Multi-Polar Spindle (MPS) formation and centrosome defects (Wang et al., 2018). Inhibiting other PARPs such as: PARP1, PARP2, PARP3, PARP5a and PARP5b, did not create the same phenotype, providing strong evidence that PARP6 plays a unique role in the regulation of MPS induction (Wang et al., 2018). Furthermore, a previous study demonstrated that PARP6 is a negative regulator of cell proliferation and that PARP6 expression leads to accumulation of cells in S-phase (Tuncel et al., 2012). While at the time the reason for this was unclear, it is likely that this was caused by PARP6s involvement in MPS induction and centrosome homeostasis (Tuncel et al., 2012; Wang et al., 2018). PARP6 expression levels have been observed to be lower in colorectal cancer compared to neighboring non-cancerous tissue (Qi et al., 2016). It is suggested this may be due to hypermethylation of the PARP6 promotor region (Qi et al., 2016). Additionally, this paper also found that PARP6 expression is negatively correlated to Survivin expression. Survivin is an inhibitor of apoptosis (IAP) family member (Jaiswal et al., 2015). This anti-apoptotic property is largely suggested to be why high expression of Survivin is correlated with cancer and tumourigenesis, implicating PARP6 as a tumour suppressor (Jaiswal et al., 2015). However, a later paper, suggested that PARP6 positively regulates Survivin in gastric cancer, with higher expression of PARP6 showing a strong correlation with increased carcinogenic cell properties including: motility, proliferation, migration and invasion (Sun et al., 2018). These studies suggest contradicting roles for PARP6 in the regulation of Survivin and this may be explained by PARP6 having different regulatory roles in different tissue types. However, further research is required to confirm this. A potent PARP6 inhibitor, AZ0108 has been found to selectively inhibit PARP6 catalytic activity (Wang et al., 2018). This inhibitor has been found to induce MPS error-induced apoptosis in breast cancer cells *in vitro* and inhibition of xenograft tumour growth *in vivo* (Wang et al., 2018). Additionally, via mass spectrometry it was found that Checkpoint Kinase 1 (Chk1) (a protein involved in the regulation of the cell cycle and DNA damage response) is a substrate of PARP6. Inhibiting PARP6 activity with AZ0108 leads to an increase in Chk1 phosphorylation and defects in mitotic signalling (Wang et al., 2018). This provides strong evidence that it could be worthwhile investigating targeting PARP6 for cancer therapy in the future.

PARP7 adds MAR modifications (Figure 2; Table 1) to its substrates and belongs to the CCCH-Zn finger PARP sub-family (Figure 3) (Vyas et al., 2013; Challa et al., 2021). It has been determined that the Zinc finger of PARP7 has a high binding affinity for RNA, suggesting a potential regulatory role in transcription (Rasmussen et al., 2021). Depletion of PARP7 leads to an increase of cells in mitosis, but not reduced viability. This suggests that cells are still able to undergo mitosis, but that mitosis progresses more slowly in the absence of PARP7 (Vyas et al., 2013; Vyas et al., 2014). PARP7 has also been shown to be a regulator of innate immunity, transcription factor activity and stress responses (Xue et al., 2018; Palavalli Parsons et al., 2021; Rasmussen et al., 2021). A recent study has shown that PARP7 is a suppressor of aryl hydrocarbon receptor (AHR) and a positive regulator of Liver X Receptors (LXRs), type I interferons (IFN-Is), and hypoxia-inducible factor I (HIF-1a), suggesting it may have a role in innate immunity. AHR, LXRs, IFN-Is and HIF1a have all been shown to have a direct link to tumourigenesis (Lin and Gustafsson, 2015; Jun et al., 2017; Xue et al., 2018; Aricò et al., 2019; Rasmussen et al., 2021). PARP7 expression levels are typically increased in a wide range of cancers, such as: colorectal cancer, head and neck cancer, liver cancer and myeloma (Cheng et al., 2019). Whereas low PARP7 expression levels were found in: bladder cancer, cervical cancer, esophageal cancer, leukemia, lung cancer, lymphoma, melanoma, and in particular breast cancer (Cheng et al., 2019). Notably, high PARP7 expression levels in breast cancer have been correlated with improved patient outcome and patients with advanced breast cancer have very low expression of PARP7 (Cheng et al., 2019). Additionally, study of the PARP7 catalytic domain suggests it plays a regulatory role in microtubule control via MARylation modifications. One study found that mutation of the PARP7 catalytic site led to an overall increase in microtubule stability, resulting in slowed growth and migration of ovarian cancer cells (Palavalli Parsons et al., 2021). A recent study identified a potent and selective inhibitor of PARP7, RBN-2397. This compound has shown promising results in lung cancer xenografts, causing tumour regression after treatment (Gozgit et al., 2021). A phase 1 clinical trial on metastatic or advanced-stage solid malignant tumours is underway to assess its efficacy. In addition, another phase 1 clinical trial is underway to examine the efficacy of RBN-2397 against advanced squamous non-small cell lung carcinoma in combination with immunotherapy, highlighting the potential of PARP7 as a cancer therapy target.

PARP8 is catalytically capable of producing MAR modifications (Figure 2; Table 1) and at present is not catagorised into any structural sub-classification (Figure 3) (Hottiger et al., 2010). PARP8 is primarily localised on the nuclear envelope for the majority of the cell cycle but localises to centrosomes and spindle poles during mitosis. Consistent with this, depletion of PARP8 is associated with mitotic and nuclear morphology defects and a decrease in cellular viability, although the mechanism behind this is unknown (Vyas et al., 2013; Vyas et al., 2014; Challa et al., 2021). To date the biological pathways PARP8 is involved in have not been uncovered. Structural modelling and experimental analysis have revealed that PARP8 has MARylation activity, although its substrates have not been identified (Hottiger et al., 2010; Vyas et al., 2014; Challa et al., 2021). To date, a cellular function for PARP8 has not been established and no PARP8 inhibitors have been investigated for anti-cancer activity or clinically developed.

PARP9 was originally suggested to be catalytically inactive due to it’s inability to undergo auto-ADP-ribosylation (Vyas et al., 2014), but it was subsequently confirmed to have MAR activity (Figure 2; Table 1) (Yang et al., 2017). Similarly, to several other PARPs, PARP9 contains macrodomains that bind ADPr and PAR (Figure 3). In addition to its proposed role in DNA repair, a recent study showed a function for PARP9 in the detection of RNA viruses (Xing et al., 2021). PARP9 has also been implicated in chemoresistance in prostate cancer and diffuse large B cell lymphoma (Camicia et al., 2013; Bachmann et al., 2014). The levels of PARP9 are also elevated in breast cancer and its depletion inhibited the migration of breast cancer cells (Tang et al., 2018). To the best of our knowledge, no PARP9 inhibitors have been identified or investigated, to date.

PARP10 is a MAR transferase (MART) (Figure 2; Table1) (Kleine et al., 2008; Vyas et al., 2013; Challa et al., 2021). PARP10 is not structurally categorised in any PARP family sub-classifications (Figure 3). Up to 70 substrates have been identified for PARP10 (Feijs et al., 2013), however, it is unclear how many of these are genuine substrates *in vivo*. A subsequent study also showed that PARP10 also promotes cellular transformation, proposed to be through the alleviation of replication stress (Schleicher et al., 2018). Supporting this assertion, PARP10 depletion significantly inhibited tumour growth in a mouse xenograft model (Schleicher et al., 2018). A novel PARP10 inhibitor, A82-(CONHMe)-B354, has recently been developed (Lemke et al., 2020). This inhibitor was found to have an IC50 of 6.0 uM via a histone ADP-ribosylation assay. This study also generated a screen of various proposed PARP10 inhibitory molecules using a PARP10 virtual combinatorial library (VCL) (Lemke et al., 2020). These proposed inhibitors require further study to determine their efficacy against tumour cells and whether clinical development would have therapeutic applications (Lemke et al., 2020).

PARP11 is catalytically capable of producing MAR modifications, but has not been assigned to a structural sub-category (Figure 3; Table 1) (Hottiger et al., 2010). PARP11 is primarily located at nuclear pores, where it co-localises with Nucleoporin153 (NUP153) (Vyas et al., 2014; Meyer-Ficca et al., 2015). PARP11 is important in cellular processes such as maintaining nuclear envelope stability and nuclear remodeling during spermatogenesis (Vyas et al., 2014; Meyer-Ficca et al., 2015) and its activity is essential for spermatid formation in mice (Meyer-Ficca et al., 2015). The silencing of PARP11 resulted in deformed sperm heads due to improper nuclear envelope formation during spermatogenesis, leading to infertility (Meyer-Ficca et al., 2015). A recent study found that ITK7 is a potent and highly selective inhibitor of PARP11 activity (Kirby et al., 2018). Inhibition resulted in disassociation of PARP11 from the nuclear envelope. Further study is needed to establish a clinical application for this potent inhibitor (Kirby et al., 2018).

PARP12 produces MAR modifications (Figure 2; Table 1) on target proteins and belongs to the Zinc Finger CCCH Domain-Containing Protein sub-family (Figure 3) (Shao et al., 2018; Challa et al., 2021). This protein is localised in the Golgi and is punctate in the cytoplasm during interphase (Vyas et al., 2013; Vyas et al., 2014; Buch-Larsen et al., 2020). There is evidence to support a role for PARP12 in the cellular stress response, through a PARP1-dependent pathway. Following oxidative stress PARP12 is translocated from the Golgi to stress granules via a mechanism dependent upon PARP1 activity (Catara et al., 2017). It is hypothesised that PARP12 may have a function in Golgi maintenance under normal cellular conditions and is required to prevent translation under stress conditions. PARP12 may also have a tumour suppressor function and supporting this, low PARP12 expression levels are associated with tumourigenesis (Shao et al., 2018). One study demonstrated that PARP12 depletion via *in vitro* CRISPR-Cas9 modification in QGY-7703 and Huh7 cells promoted liver cancer cell migration (Shao et al., 2018). This was further supported by an *in vivo* metastasis assay that showed that PARP12 deficiency in mice promoted hepatocellular carcinoma metastasis via the regulation of the epithelial-mesenchymal transition process (Shao et al., 2018). To date, no studies have reported PARP12 selective inhibitors. Given the roles PARP12 plays in tumourigenesis and maintaining cellular homeostasis, identifying selective inhibitors of its activity may be an effective therapeutic strategy for cancer treatment.

PARP13 has no defined catalytic activity, producing neither PAR nor MAR modifications. However, its structure suggests that it is capable of producing MAR modifications (Table 1), although this has yet to be experimentally demonstrated (Hottiger et al., 2010). PARP13 belongs to the Zinc Finger CCCH Domain-Containing Protein sub-family (Figure 3) and is localized to punctate structures throughout the cell during interphase and is punctate in the cytoplasm during mitosis (Vyas et al., 2013; Vyas et al., 2014; Buch-Larsen et al., 2020; Challa et al., 2021). Depletion of PARP13 has a strong negative impact on cell viability, although the reason for this has not been determined (Vyas et al., 2013; Vyas et al., 2014). PARP13 is involved in specific anti-viral pathways, including recruiting cellular RNA degradation machineries such as poly(A)- specific ribonuclease (PARN) that removes the poly A tail of the viral mRNA (Todorova et al., 2014; Todorova et al., 2015). To date no PARP13 inhibitors have been reported and the impact of its depletion on tumour cell growth has not been investigated.

PARP14 produces mono-ADP ribosylation (MAR) modifications (Figure 2; Table 1) on target proteins (Vyas et al., 2013; Vyas et al., 2014; Buch-Larsen et al., 2020; Challa et al., 2021). PARP14 is associated with a multitude of disease states, including; cancer, atherosclerosis and the inflammatory response to allergens (Qin et al., 2019). PARP14 is an actin cytoskeleton-regulating, Macro-domain containing PARP (Figure 3) (Vyas et al., 2013; Buch-Larsen et al., 2020; Challa et al., 2021). Depletion of PARP14 leads to actin cytoskeletal defects, and overall cell viability defects. One study found that PARP14 depletion caused a phenotype with elongated processes extending from the cell body in approximately 60% of siRNA transfected cells (Vyas et al., 2013). It was hypothesised that this is due to the cells inability to retract and dismantle actin filaments as the cell moves. This provides strong evidence that PARP14 is important in maintaining cytoskeletal structure and cell motility. In addition to cytoskeletal regulation, PARP14 regulates the expression of B-cell survival factors and represses caspase apoptotic pathways to transduce survival signals in murine primary B cells (Cho et al., 2011; Barbarulo et al., 2013). This implicates PARP14 in promoting tumourigenesis via its role as a downstream effector of JNK2 and inhibiting the JNK1-JNK2 pro-apoptotic pathway (Barbarulo et al., 2013). In support of targeting PARP14 to treat cancer, an inhibitor of PARP14, RBN012759, was shown to lead to an inflammatory response in tumour explants, similarly to that induced by immune checkpoint inhibitors (Schenkel et al., 2021). This compound inhibits PARP14 activity at very low concentrations and displays approximately 300-fold selectivity for PARP14 over other highly homologous PARP family members (Schenkel et al., 2021). This makes targeting PARP14 a promising avenue for developing cancer therapeutics (Schenkel et al., 2021). However, further research needs to be conducted to fully elucidate the mode of action and clinical application of this inhibitor.

PARP15 is a catalytically active PARP that produces MAR modifications (Figure 2; Table 1) and is a member of the macro-PARP subfamily (Figure 3) (Vyas et al., 2013; Challa et al., 2021). PARP15 has low protein expression levels in cells and therefore its localisation and the effects of its depletion are unknown (Vyas et al., 2013). Two single nucleotide polymorphisms (SNPs) of PARP15 (*rs6793271, rs17208928*) have been associated with decreased survival rates in patients with acute myeloid leukemia (Lee et al., 2016). Further study is needed to elucidate whether PARP15 is involved in tumourigenesis and if it would be an appropriate target for tumour therapy. To date, no selective inhibitors of PARP15 have been reported.

PARP16 produces MAR modifications (Figure 2; Table 1) and is the smallest member of the PARP super-family (Table 1) (Hottiger et al., 2010). Notably, PARP16 possesses a tail anchor for attachment to membranous structures, which is a unique characteristic within the PARP protein family and such PARP16 is not categorized into any of the other structural sub-families (Figure 3) (Vyas et al., 2013; Yang et al., 2020). During interphase it has a punctate localisation and is within the membrane of the endoplasmic reticulum (ER); during mitosis it is punctate in the cytoplasm (Vyas et al., 2013; Vyas et al., 2014; Buch-Larsen et al., 2020). Depletion of PARP16 is associated with a defective membrane phenotype, with 30% of cells exhibiting completely round cell membrane morphology, suggesting a role in membrane structure. GFP-tagged PARP16 has been observed to localise to the ER membrane (Vyas et al., 2013), further supporting that PARP16 is involved in the maintenance or formation of the ER membrane. Failure to maintain proteostasis due to decreased ER efficiency is considered a driving factor of cellular aging and cancer. PARP16 also positively regulates ER stress sensors (PERK and IRE1) during the unfolded protein response (UPR), which is associated with cellular senescence (Yang et al., 2020). As such, inhibition of PARP16 activity in Angeotensin II (Ang II)-treated mice and vascular cells was found to reduce senescence-associated phenotypes (Yang et al., 2020). Ang II plays a key role in regulating the renin-angiotensin system (RAS), an increase in Ang II causes an increase in blood pressure (Benigni et al., 2010). Due to the critical role of PARP16 in the cellular stress response, it has been speculated that PARP16 maybe an efficient cancer target. Supporting this, treatment of a hepatocellular carcinoma cell line with a small molecule inhibitor of PARP16 in combination with agents that induced ER stress led to enhanced apoptosis (Wang et al., 2017). Further investigation of PARP16 inhibitors is required to explore the utility of their use as a cancer therapeutic. PARP16 has been identified as a potent novel target for cancer therapeutics when inhibited in conjunction with PARP1. For example, silencing PARP16 *in vitro* reduced cancer cell survival when cells were treated with the PARP1 inhibitor Olaparib and the WEE1 inhibitor adavosertib (Palve et al., 2021). In addition, chemical proteomics identified PARP16 as a novel secondary target of PARP inhibitor talazoparib. This raises the possibility that the off-target inhibitory effects of talazoparib on PARP16 may contribute to its potency as a selective cancer therapeutic and may support targeting PARP16 as an anti-cancer therapy (Palve et al., 2021).

## PARP Protein Involvement in the DNA Damage Response

DNA Damage repair, and genetic instability are intrinsically linked as hallmarks of cancer. Many members of the PARP protein family have been found to have strong involvement in these pathways. In contrast, many others are still yet to have their functional involvement in DNA damage fully elucidated.

In terms of a potential role for PARP4 in DNA damage, one study examined whether vault proteins relocalised to UV-induced DNA damage and found that PARP4 did not respond to UV irradiation (Kickhoefer et al., 1999). It is possible that PARP4 may respond to other forms of DNA damage, but to date no other experimental evidence has directly implicated PARP4 in DNA damage repair processes. However, PARP4 does contain a BRCT domain (Perina et al., 2014), which is prevalent in many DNA repair proteins, including PARP1, which may support a role for PARP4 in DNA repair and tumourigenesis (Jean et al., 1999; Perina et al., 2014; Hu et al., 2019).

There is currently no literature supporting a role for PARP6 in the DNA damage response and further study is required to establish what, if any, role it has in this process.

A direct role for PARP7 in DNA repair has not been confirmed experimentally, however a recent study detected several DNA repair proteins (including PARP1, and PARP2) as substrates of PARP7 MARylation, suggesting that PARP7 may have a regulatory role in DNA repair (Palavalli Parsons et al., 2021). PARP7 also undergoes auto-PARylation and has been shown to have affinity for PARP4 as a substrate (Palavalli Parsons et al., 2021). Auto-PARylation is heavily associated with DNA Damage repair related PARPs such as: PARP1. Moreover, the CCCH-type Zinc Finger of PARP7 suggests it may have a high binding affinity for RNA, raising the possibility that PARP7 could be a potential regulator of transcription or RNA-dependent DNA repair. However, more studies need to be conducted to establish these potential roles.

Supporting a role for PARP8 in DNA repair, auto-PARylation of a PARP8 cysteine residue occurs in response to oxidative stress induced by H_2_O_2_ treatment, suggesting that, like PARP1, it may have a role in the oxidative stress response (Buch-Larsen et al., 2020). Further investigation is needed to establish the precise function of PARP8 in the DNA damage response.

PARP9 has been shown to interact with the ubiquitin (Ub) E3 ligase Dtx3L to form a heterodimeric complex, which mediates mono-ubiquitylation of Histone H4 following DNA damage (Camicia et al., 2013; Yan et al., 2013). Expression of GFP-tagged PARP9 macrodomains were shown to be recruited to sites of DNA damage induced by microirradiation, suggesting that PARP9 is likely to have a direct role in the DNA damage response (Yan et al., 2013). Cells depleted of PARP9 or Dtx3L were also shown to have a 50% decrease in DNA double strand break repair via non-homologous end-joining (NHEJ), suggesting that the Dtx3L/PARP9 complex has a direct role in DNA repair (Yan et al., 2013; Yang et al., 2017). However, PARP9 deficient mice were subsequently shown to have functional V(D)J, which requires NHEJ, suggesting that PARP9 may not be essential for NHEJ or that compensatory mechanisms are involved (Robert et al., 2017). Taken together, the roles of PARP9 in DNA repair and tumourigenesis suggest it may be a successful anticancer target, however further investigation is required.

Like several other members of the PARP protein family, depletion of PARP10 results in genomic instability and hypersensitivity to DNA damaging agents. It was also demonstrated that PARP10 has a role in DNA repair and cooperates with the replication-associated Proliferating Cell Nuclear Antigen (PCNA) to mediate translesion synthesis in response to UV-induced lesions (Feijs et al., 2013; Nicolae et al., 2014). A subsequent study also showed that PARP10 also promotes cellular transformation, proposed to be through the alleviation of replication stress (Schleicher et al., 2018). Supporting this assertion, PARP10 depletion significantly inhibited tumour growth in a mouse xenograft model (Schleicher et al., 2018). Given that PARP10 has a role in DNA repair and its depletion inhibits tumour growth, it is suggested that it may be a chemotherapeutic target.

Further study needs to be conducted to determine if PARP11 has a role in DNA damage response pathways and tumourigenesis, to date its roles in these processes have not been established.

The direct involvement of PARP12 in the DNA damage response is yet to be experimentally confirmed. However, its PARP1-dependent response to cellular stress may indicate that it plays a role in the DNA damage response, although further study is required to fully elucidate the roles of PARP12 in this process.

In addition to antiviral responses, PARP13 has also been shown to be a mediator in DNA damage repair (Todorova et al., 2015; Fujimoto et al., 2017) and forms a complex with PARP1 and heat shock transcription factor 1 (HSF1). This complex aids in the facilitation of DNA damage repair via transportation of PARP1 (Fujimoto et al., 2017), which then disassociates from this complex and localises to sites of DNA breaks to promote repair. It is likely that it is via this pathway PARP13 plays a tumour suppressive role. Additionally, one paper hypothesised that the inhibitory effect of PARP13 on TRAILR4 (pro survival receptor) sensitises cells to TRAIL mediated apoptosis, acting as a protective barrier against tumourigenesis (Todorova et al., 2015). For these reasons PARP13 may be a strong novel target for designing cancer therapeutics.

PARP14 has been shown to have a role in DNA repair via an interaction with PCNA at replication forks promoting replication of DNA lesions and fragile sites (Nicolae et al., 2015). Depletion of PARP14 also leads to a decrease in repair of double-strand breaks via homologous recombination and subsequent sensitivity to DNA damaging agents such as bleomycin and hydroxyurea (Nicolae et al., 2015). The homologous recombination repair protein Rad51 was shown to be MARylated by PARP14. Furthermore, PARP14 also contains a Macro2 domain which enables it to recognise and bind MARylated substrates including Rad51 (Nicolae et al., 2015). This supports a direct role for PARP14 in DNA repair. A link was also observed between high PARP14 expression levels and poorer prognosis in multiple myeloma (Cho et al., 2011; Barbarulo et al., 2013; Iansante et al., 2015; Dhoonmoon et al., 2020). Moreover, another study found that PARP14 promoted cancer cell proliferation in hepatocellular carcinoma by promoting the Warburg effect (Iansante et al., 2015). In light of these studies, it has been suggested that PARP14 may be a novel drug target for several cancer types including diffuse large B-cell lymphoma, multiple myeloma prostate cancer and hepatocellular carcinoma (Qin et al., 2019). The role of PARP14 in DNA repair also suggests its inhibition may sensitise tumours to DNA-damaging chemotherapeutics (Qin et al., 2019).

The involvement of PARP15 and PARP16 in DNA damage repair has not been experimentally shown to date, further research needs to be conducted to determine this.

## Conclusion

Studies of PARP family proteins have primarily focused upon PARP1-3 and the Tankyrases. As such, the roles of these prevalent proteins have been well defined in DNA repair, telomere maintenance, tumourigenesis and cancer therapy. In contrast, far less is known about the rest of the PARP family proteins. Here, we have highlighted the diverse and intricate roles the PARP family play within the cellular environment to maintain cellular homeostasis. Given that several of the PARPs discussed here have potential roles in mitosis and DNA repair, it is likely that the other PARP proteins could represent future targets for cancer therapy. The role of these PARPs in DNA repair and cell division may form the focus of subsequent studies and guide the consensus to develop further PARP family members as targets of anti-cancer therapy.
